# Mother-newborn couplet care: Nordic country experiences of organization, models and practice

**DOI:** 10.1038/s41372-023-01812-3

**Published:** 2023-12-12

**Authors:** Stina Klemming, Siri Lilliesköld, Sofia Arwehed, Wibke Jonas, Liisa Lehtonen, Björn Westrup

**Affiliations:** 1 Lund-Malmö NIDCAP Training and Research Center, Department of Neonatology, Skåne University Hospital, Lund, Sweden; 2 Department of Women’s and Children’s Health, Karolinska Institutet, Stockholm, Sweden; 3 Department of Neonatology, Astrid Lindgren’s Children Hospital, Karolinska University Hospital, Stockholm, Sweden; 4 Department of Women’s and Children’s Health, Uppsala University, Uppsala, Sweden; 5 Department of Neonatology, Uppsala University Hospital, Uppsala, Sweden; 6 Faculty of Medicine, University of Turku, Turku, Finland; 7 Department of Pediatrics and Adolescence Medicine, Turku University Hospital and University of Turku, Turku, Finland

**Keywords:** Translational research, Outcomes research

## Abstract

Mother-Newborn Couplet Care is a concept and is defined as the provision of care for a sick or preterm newborn in close proximity to and coupled with the care for the mother from the birth of the infant and for as long as the mother needs hospital care. This concept of care requires system change in both obstetrics and pediatrics in terms of the planning and organization of care, equipment and design of units. Accordingly, strong leadership setting clear goals and emphasizing a culture of cohesive care, supported by targeted education and training is crucial to ensure high-quality care of all mother-newborn dyads without separation. We describe various organizational models of Mother-Newborn Couplet Care used in Sweden and Finland and implementation processes. We envision a future where newborns and mothers are always together, irrespective of medical needs, and form an inseparable center around which healthcare services and providers are organized.

## Rationale and evidence

**Fig. 1:**
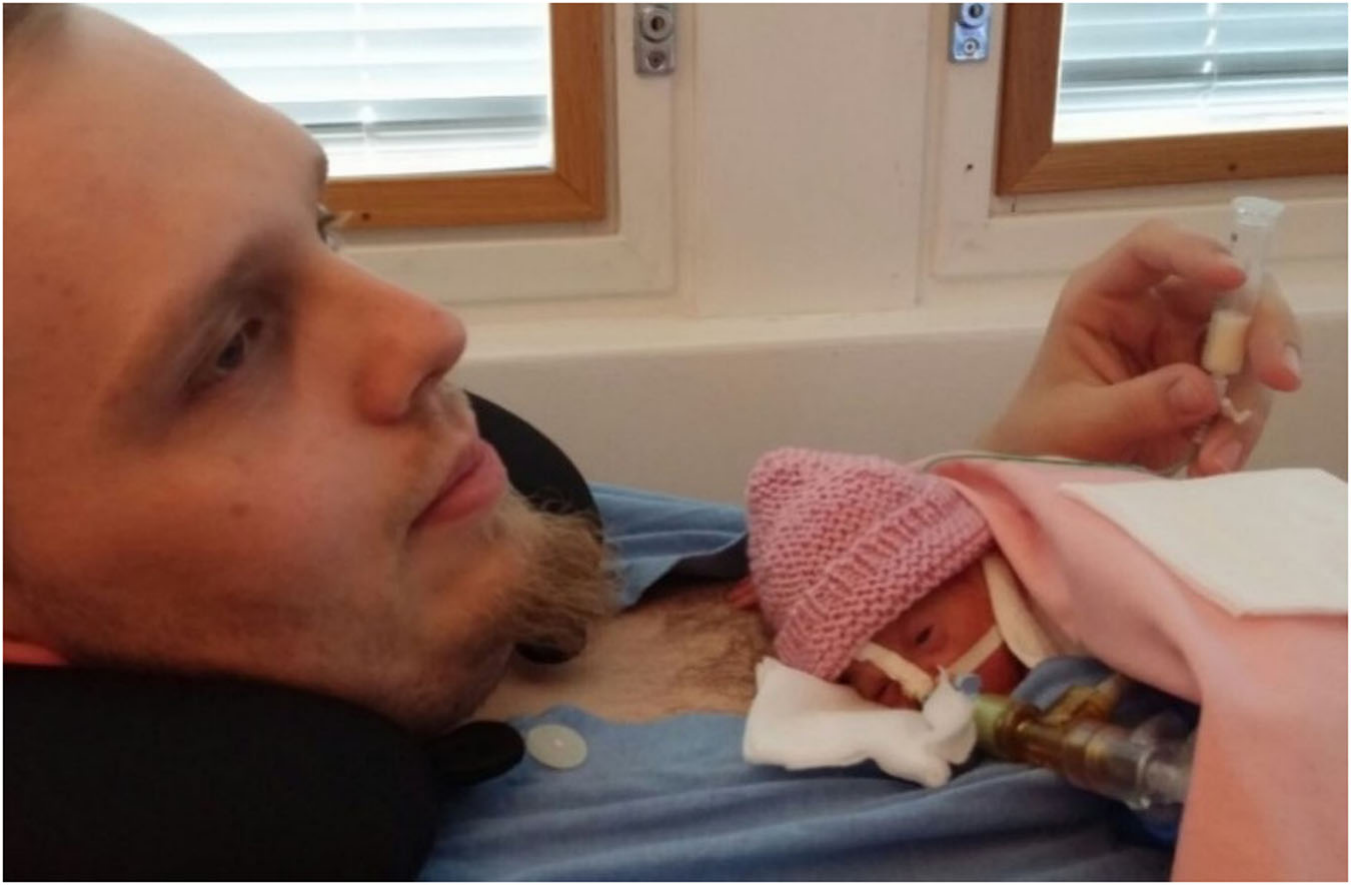
An extremely preterm infant in the neonatal intensive care unit, receiving mechanical respiratory support in skin-to-skin contact with the father. Photo: The NICU at Turku University Hospital.

Being nurtured, loved, and cared for by the parents from the very moment of birth is what the newborn expects and needs to begin the journey of unlocking his or her full potential. This is true for all infants, including those in need of medical care [1]. While the natural condition is for a newborn infant to stay in close physical contact with their mother after birth, separation is still common when newborns need specialized care [2]. There is however, an increasing awareness of and evidence for the importance of keeping parents and infants together after birth and integrating medical technology and pharmaceutical treatments with caregiving that ensures parent-infant closeness [3–6]. The vision of *non-separation* of the mother-newborn dyad is stressed in the new World Health Organization (WHO) recommendations for care of the preterm or low-birthweight infant and supported by several meta-analyses. The WHO makes a strong recommendation for skin-to-skin contact (or Kangaroo Mother Care, KMC) to be started “as soon as possible after birth, before the infant is clinically stable, unless the infant is unable to breathe spontaneously after resuscitation, is in shock or needs mechanical ventilation” [7]. In high-resource settings skin-to-skin contact is often applied also during mechanical ventilation soon after birth (Fig. 1). Furthermore, the WHO recommends as much subsequent continuous skin-to-skin contact as possible, or at least 8 h per day. The WHO additionally recommends the implementation of couplet care by providing infrastructure, equipment and supplies needed for medical and nursing care for both the mother and a preterm, low-birthweight or sick newborn in a way that enables being together from the birth [8, 9].

Recently, the immediate KMC Study, a large multinational randomized controlled trial (RCT) in low- and middle-income countries demonstrated a mortality reduction of 25 percent in the group of immediate skin-to-skin contact and non-separation compared to conventional care in very low birth-weight infants [6, 10]. Several trials in high-resource settings have demonstrated the benefits of keeping mothers and their infants physically and emotionally close even after very preterm or complicated births. A recent RCT in Sweden and Norway reported that immediate skin-to-skin contact for very preterm infants led to more stable infant physiology and positive and meaningful experiences of the parents, including fathers [11–13]. Another RCT from Norway similarly reported feasibility and safety of immediate or early skin-to-skin contact for very preterm infants as well as positive experiences of the mothers [14, 15]. A RCT in Germany reported less impaired bonding and improved motor and vocal development of the infant, as well as reduced expression in studied stress-related genes in the group receiving immediate skin-to-skin contact, compared to conventional care after a very preterm birth [16, 17]. The feasibility, safety and positive parental experiences of “delivery room cuddle”, in which the mother holds her extremely preterm or sick infant for a brief period of time prior to the admission to the neonatal intensive care unit (neonatal unit), have been reported by different study groups in the United Kingdom [18–20]. Non-separation can be attained, and it is preferred by parents, also during the resuscitation of the infant if the stabilization procedure is performed near the mother in the labor ward/operating room [21–23].

According to the principles of Infant- and Family-Centered Developmental Care, hospitalized infants should be together with and be cared for by their parents, in partnership with the healthcare staff [24, 25]. The principles of nurturing care for all children and non-separation from their parents is also stated in the Convention on the Rights of the Child and in the World Association for Infant Mental Health Declaration of Infants’ Rights [26, 27]. In traditional healthcare systems, the practice of non-separation has been challenging during the first hours and days after birth and especially when both the mother and the infant are in need of medical care [28, 29]. Thus, it is crucial for medical facilities caring for newborns and mothers to reorganize and plan the care of both patients with the goal of minimizing separation for all [2]. Accordingly, in a recent Global Position Paper, the WHO presents a new vision where *“mothers, newborns and families form an inseparable center around which the entire maternal newborn service delivery is organized”* [30].

## The definition of Mother-Newborn Couplet Care

Mother-Newborn Couplet Care (couplet care) is a concept where the care for a sick or preterm newborn is provided in close proximity to and coupled with the care for the mother from the birth of the infant and for as long as the mother needs hospital care. The location for the care of the dyad is decided according to a medical assessment of each patient, usually determined by the patient with the most complex medical needs, the local context and the flexibility within the system.

## Operational and organizational models for Mother-Newborn Couplet Care

Couplet care can be structured in different ways and should be adjusted to the local context. The choice of organizational model will be influenced by many interrelated factors such as the physical structure of the units and the hospital, the organizational structure and collaboration between different departments involved in the care of the mother and the newborn as well as the organization and reimbursement system for care providers in the health region. Local and national guidelines, fiscal policies and governmental recommendations will also affect how couplet care is organized and implemented. In practice, couplet care for a sick or preterm infant is most often provided in the neonatal intensive care unit with the mother receiving her care there, provided by midwives or maternity nurses and obstetricians, in close proximity to her newborn. However, care for the two patients together could be organized in any of the units caring for a newborn and a mother. In addition to a neonatal unit, newborns can also be cared for in a pediatric cardiac or surgical unit or in a maternity ward. Depending on the mode of birth and medical complexity of the care needed by the mother couplet care can be provided in the labor ward, the maternity ward, the surgical, post-surgical and anesthetic units or in the adult intensive care unit. To guarantee patient safety, it is important that all units involved appropriately educate staff, adjust the design and structure of the unit and implement guidelines and checklists for both the infants and mothers. Based on our experiences in Sweden and Finland we have identified four different organizational models of couplet care.

### Mother-newborn dyad cared for by obstetric and neonatal teams from separate departments

Couplet care is provided in collaboration by care teams from the departments caring for either the sick or preterm infant or the mother. Midwives or maternity nurses and obstetricians from the obstetric department are responsible for the medical and nursing care of the mother and provide care for her wherever she is physically located. Likewise, neonatal nurses and neonatologists from the neonatal unit provide care for the sick or preterm infant. The need for cross-training in care procedures is limited to basic knowledge that the teams need to be able to help each other and to provide first aid care for emergency situations. Apart from the necessary adjustments due to the care team being mobile, treating the patients in various hospital premises and improving facilities for the mothers being cared for in a neonatal unit, many things can be organized traditionally - the care is simply provided in non-separation. This organizational model of couplet care is the most common in Sweden and Finland.

### Combined nursing team with midwives as part of the neonatal team

This organizational model offers a combined nursing team for the care of the mother and newborn in need of specialized care as a dyad, by having midwives or maternity nurses employed in the neonatal department as part of the neonatal team. This model builds on close collaboration with the department of obstetrics, as the overall medical responsibility for the mother stays with the obstetricians conducting daily medical rounds together with the midwives or maternity nurses in the neonatal ward. The nurses and midwives are extensively cross-trained to ensure safe care of both infants and mothers. To our knowledge, this model has been implemented in one unit in Sweden, one unit in Finland and one unit in Canada [31].

### Specialized newborn care in the maternity ward

Infants with mild and transient medical needs can often stay with their mothers in the postnatal ward instead of being admitted to the neonatal unit. Conditions where this might be possible include late preterm birth, feeding difficulties or hypoglycemia with a need of supplementary oral feeding or a feeding tube, a need for phototherapy, antibiotic administration, neonatal abstinence symptoms and observation and treatment for mild transient tachypnea. The medical responsibility for the specialized care of the infant remains with the doctors from the neonatal unit doing daily medical rounds. Most often nurses and midwives in the maternity ward care for the mother-newborn dyad. However, for more complex care in the maternity ward, like nasogastric tube-feeding and the administration of antibiotics in a peripheral line, nurses from the neonatal unit often take the nursing responsibility. When newborns with more complex medical needs are cared for in the maternity ward, midwives and nurses need special training. This model has been easy to implement in all maternity units throughout Sweden and Finland since all units implement the rooming-in concept with nursing staff trained in caring for both the mother and, under pediatric supervision, the healthy newborn.

### A department of perinatology with obstetric and neonatal care in one organization

Staff for obstetric and neonatal care are employed by a department of perinatology providing antenatal and post-partum care of the mother and the care of the newborn, regardless of the level of care required by both patients. The department of perinatology might even have its own anesthetic unit, including an adult intensive care unit. Training, competencies and care are separate for the obstetric, neonatal and anesthetic staff. However, one common department facilitates collaboration between the various medical and nursing staff members and systems and services become more flexible. The need for extensive cross-training is less prominent when all services and competencies are under the same management. This organizational model has primarily been chosen when starting a new department, and it has been implemented in two hospitals in Sweden and one in Finland.

## Mother-Newborn Couplet Care at birth and during the first hours after birth

**Fig. 2:**
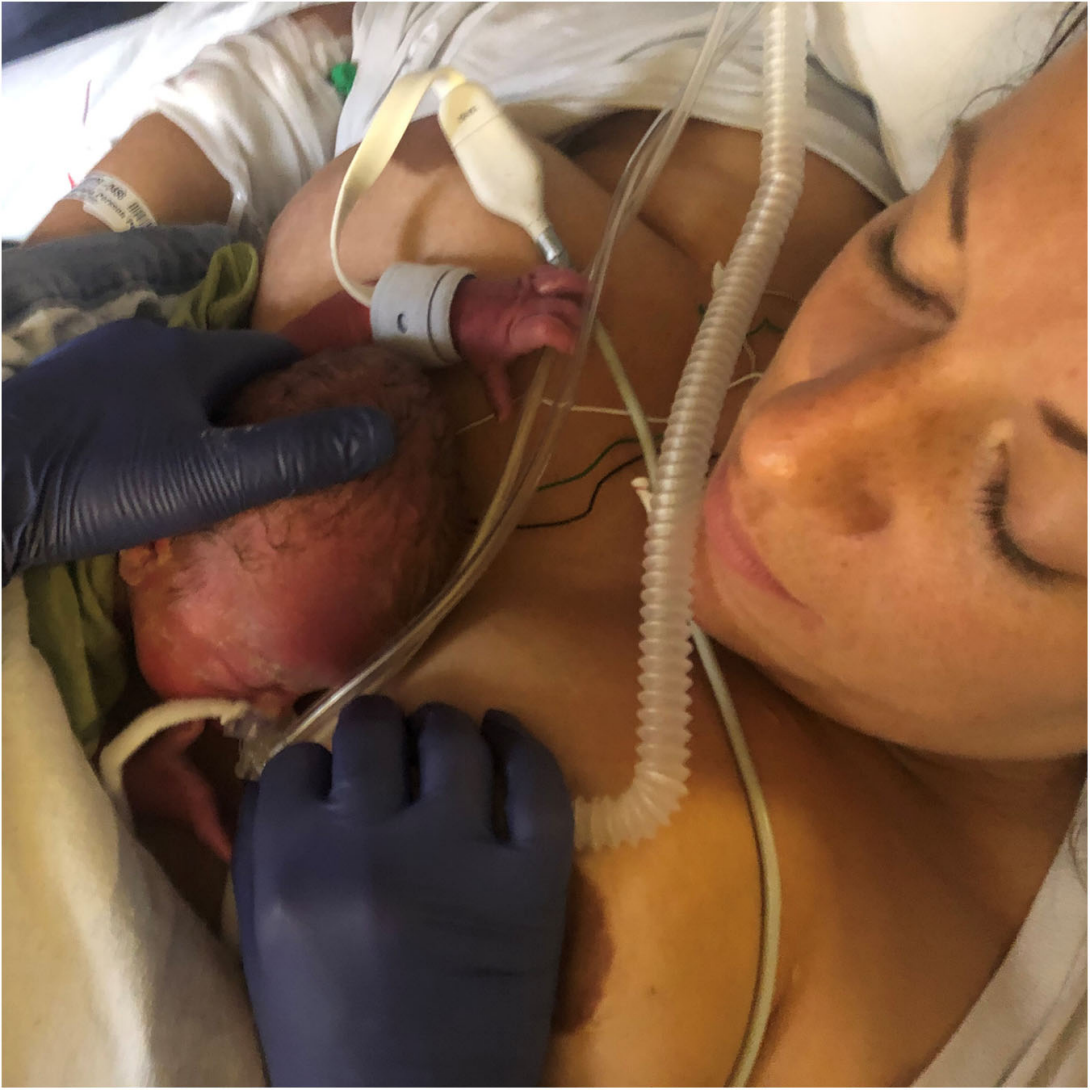
A very preterm infant receiving non-invasive respiratory support in immediate skin-to-skin contact with his mother. Photo: Stina Klemming.

**Fig. 3:**
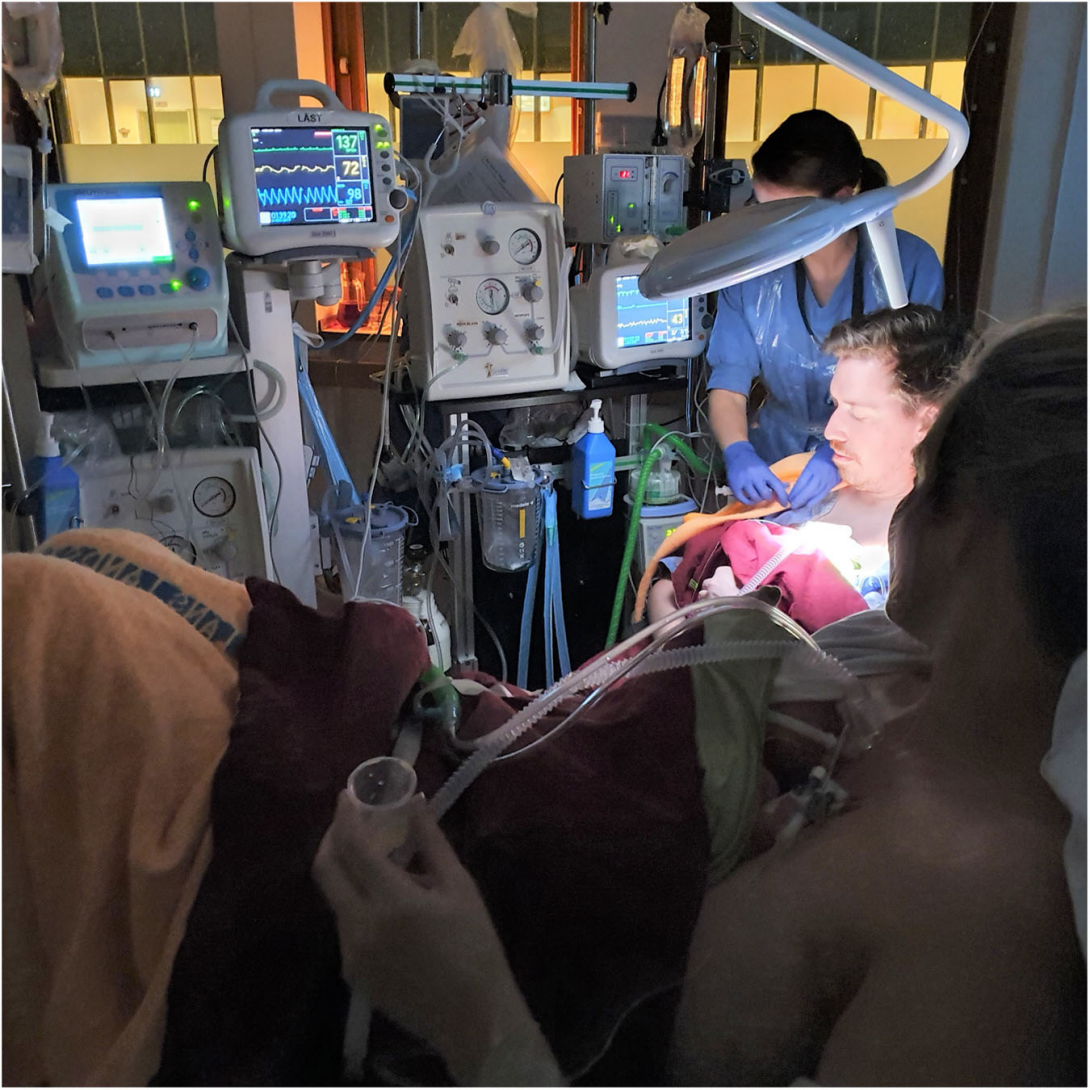
Twins born very preterm, receiving immediate skin-to-skin contact directly after birth with the mother and father respectively. A mobile neonatal team with equipment for neonatal stabilization in the birth room provide non-invasive respiratory support and iv-treatment for hypoglycaemia. Note the mother’s iv therapy and the cup for early milk expression. Photo: Stina Klemming.

Ideally, the non-separation of the mother and her newborn infant - couplet care - starts as soon as the infant is born. Depending on gestational age and the medical condition of the infant, it is possible to provide most of the caregiving and treatment without separating the infant from the mother, in her room in the labor ward or operating theatre. The neonatal team may start the care of the newborn when the newborn is positioned on the mother’s body in immediate skin-to-skin contact (Fig. 2), or alternatively on a resuscitation table or resuscitation trolley at/near the mother’s bedside. The birth room should be spacious so that all necessary equipment can be readily available and prepared well in advance, and the team should be mobile for safe and flexible care. Physical proximity between the mother and her infant facilitates close collaboration and communication between the team assisting the birth and the team preparing for and caring for the newborn, as well as with the mother. Mobile equipment bedside also facilitates stabilization of the infant with sustained cord circulation during the first minutes after birth and implementation and evaluation models have been described [32, 33]. A mobile neonatal team can continue to provide care for the newborn in the same room where the mother is cared for (Fig. 3). The care may most optimally be provided while the mother and infant are placed in skin-to-skin contact until they are transferred together to the neonatal unit. If the condition of the infant requires immediate transfer, the mother will follow her infant to the neonatal unit, where maternal postpartum care is provided. Mobile equipment enables safe and flexible care of the infant in the birth/operating room or post-operative room or even the adult intensive care unit, as well as during the transfer of the mother-newborn dyad. The mobile devices can be designed to enable uninterrupted skin-to-skin contact with the mother or the other parent during transfer. A postpartum risk assessment of the mother and the newborn respectively can help to tailor care for optimized safety by determining their needs for level of care and place of care. Many mothers to infants in need of neonatal care will be assessed as low risk patients and can be transferred to the ward where her infant needs to be cared for soon after giving birth. As the experience and robustness of the couplet care system evolves, mothers with more complex medical needs can receive couplet care in the neonatal unit.

## Planning and implementation processes

Staff attitudes, values and professional approaches lay the foundation for ways of working, enabling care for newborns and their mothers as a dyad. This can only be reached through the commitment of the leadership together with the staff of all departments involved and with the support of the hospital management. In many settings, couplet care has been achieved with a well-planned stepwise implementation process, rather than by a full implementation overnight in a new mother-newborn unit. However, it is crucial that the leaderships of the hospital and departments stay united in achieving the vision of non-separation of the mother and newborn and, subsequently, impose focused management that steadily leads the development in this direction. National guidelines and recommendations by governmental health institutions as well as organizational and financial support from hospital management are helpful and warranted although not necessary when implementing the new concept.

### Leadership

To effectively manage the process of establishing and improving couplet care, the leadership role and structure need to be considered. Depending on the couplet care organization, leadership can be structured in different ways. From our experience, the implementation and continuous assessment of the couplet care model is facilitated by creating specific leadership groups.

A leadership steering group that includes the heads of all departments involved in the care of the mother and infant (labor, postpartum, operating, anesthesiology, and neonatology/pediatric units) facilitates communication and gives a strong decision-making mandate regarding larger investments, priorities at hospital level, and changes in the work environment. The leaders of departments have an important role in communicating vision, goals, and long-term commitment to implementation. The leadership steering group also defines, follows and reports on quality indicators. The evaluation of medical safety for the mother and infant naturally runs parallel with the planning and implementation process. Moreover, it is crucial that these follow-up data are effectively communicated both up- and downstream in the organization. It is also the responsibility of this group to anchor the decisions and priorities with the hospital administration, and preferably also with the family representatives and politicians, to ensure that the importance of sustainable couplet care is understood.

A project group with managers of all wards involved, local leaders of all professions engaged and family representatives, can plan and execute the detailed process to identify each unit’s individual needs for improvement, provide evidence-base for and communicate stepwise actions for change as well as support follow-up and facilitate changes and adjustments as needed in the daily clinical work. Senior medical advisors and local “champions” within each profession and each department have important roles in supporting the project group in safety evaluation and communication.

### Additional resources and cost-benefits

The introduction of couplet care often necessitates extra investments, such as training of staff, new equipment and possibly reconstruction of facilities in the neonatal unit to make the mothers’ care possible. On the other hand, couplet care in the neonatal unit has a potential to improve the quality of care, demonstrated by reduced morbidity and mortality and improved long-term outcomes, as well as to improve care satisfaction and well-being for parents and infants. Thereby, couplet care will save costs through increased efficiency of care and reduced healthcare costs, which has been reported in a systematic review, randomized control trials and a retrospective mediation analysis [6, 10, 30, 34–41]. Other potential financial savings come from the reduced need of resources and time, like reduced floor space need when the mother and the infant share the same room, fewer transportations of the mother between wards and improved collaboration of the staff from different departments, which fit the lean framework principles.

### Reimbursement and health management information systems and follow-up

Depending on the reimbursement system of a hospital, contracts between different departments may be needed to cover the expenses for staff, medication and premises when staff provide care for patients in novel locations. As part of the routine health systems evaluation, relevant data related to couplet care include length of stay for mothers and newborns in the different locations, skin-to-skin contact time, breastfeeding, infections and pulmonary diseases in the newborn, maternal plasma hemoglobulin at discharge, need of antihypertension treatment for the mother, parental stress and mental health indicators and long-term child development for vulnerable infants [6, 10, 31, 34, 35, 39, 41–46]. Implementation research is recommended to identify, address and report context-specific challenges. It would inspire others to allow local sociocultural adaptations and ensure a continuous refinement of the implementation process [38].

### Cross-training of staff

Although the medical and nursing responsibilities are clearly divided between the staff for obstetric, neonatal and anesthetic care also in couplet care, the exchange of knowledge and clinical guidelines between the respective specialties is recommended. The development of education plans adapted to the local context could include hands-on training in caregiving skills; supporting early and continuous skin-to-skin contact, breastfeeding and early bonding and attachment processes; as well as handling maternal or neonatal emergency situations. For example, even if a mother in the neonatal unit is cared for by staff from the obstetric department, the neonatal staff should also be able to recognize acute postpartum medical complications. Likewise, when the infant’s medical condition permits couplet care to be provided in the birth room, the post-operative ward or the maternity ward, the nurses and midwives need new knowledge and skills in neonatal care. Furthermore, when planning to implement couplet care, visits to hospitals which are already using it, mockups and simulations have been reported to be helpful for the evaluation of medical safety, engaging staff, raising awareness, and creating mutual understanding of the possibilities and goals of the local model [31]. In addition, short rotations for staff members into the counter department have been found to be valuable.

## Unit design with focus on closeness and safety

Adjustments of the design of the units might be needed to ensure effective and safe couplet care that facilitates closeness between the mother and the newborn. The units involved must not only provide facilities enabling the mother to stay with her infant 24 h a day, but also provide safe care for both patients, including mothers with complex medical conditions after birth. Both the American NICU Design Standards and the European Standards of Care for Newborn Health on NICU Design provide comprehensive and detailed design recommendations [47, 48]. The equipment, medications and examination spaces required for both the routine and emergency care of mothers during the postpartum period and for infants are essential. Obstetricians and midwives or maternity nurses, as well as neonatal nursing and medical staff must be easily reached by phone, direct call, or alarm systems, and readily available in the case of rapid deterioration of the medical condition of a mother or an infant. Access to the operating room needs to be easy, and all doors in the neonatal unit need to be wide enough for an adult hospital bed on wheels to pass. The mother and infant should be cared for within a shared space that allows emotional and physical closeness, including skin-to-skin contact and helps the mother to participate in the care of her infant. If a single-family room cannot be provided, arrangements could be made to increase privacy by using screens, for example. A hospital bed is recommended for the mother for hygienic reasons and because it improves ergonomics for both the staff and the mother. The bed for the mother should have a removable headboard in the case of an emergency situation. Rails on the bed are recommended for the safe sleeping of the mother and the infant and for long periods of skin-to-skin contact. The father or the other parent should have the possibility to be with the infant 24/7. For logistic flexibility it is recommended to have similar and interchangeable hospital beds for both parents. The sound and light environment in a neonatal unit should be planned to safeguard sleep for parents and infants [40]. The parents may need a separate bedroom if the infant receives high-intensity care. Taking care of mothers in the neonatal unit also creates a need for sanitary facilities such as (private) bathrooms with showers, as well as arranging the provision and logistics of the mothers’ meals.

## Couplet care as a component of Infant- and Family-Centered Developmental Care

The recognition of the perspectives of the infant and the parents is fundamental to understanding the importance and the imperative priority of always keeping mothers and newborns together. Training and guidance in Infant- and Family-Centered Developmental Care (IFCDC) have been shown to change the attitudes and values of staff. IFCDC training is a driving force to develop parent-infant closeness with sensitive individualized care based on infant behavior, partnership with parents as primary caregivers and support and facilities for families staying with hospitalized newborns 24 h per day [49, 50]. In addition, this has facilitated the incorporation of non-separation of mothers and newborns and implementation of couplet care [51–53]. In Sweden, the implementation of couplet care was facilitated by education in the Newborn Individualized Developmental Care and Assessment Program (NIDCAP), starting in the early 1990s [54]. NIDCAP-based care has since been implemented throughout Sweden, and almost half of the Swedish units include active NIDCAP professionals. In addition, starting from 2016, a majority of the Swedish neonatal units currently implement the NIDCAP foundational educational program, Family- and Infant Neurodevelopmental Education (FINE), to mentor their staff in IFCDC [55]. In Finland twelve out of the 23 maternity hospitals have carried out a structured training with the program Close Collaboration with Parents, either for the neonatal staff or both the obstetric and neonatal staff [52]. In addition to the shown improvements in the development of IFCDC, many managers of the neonatal units in Finland reported that the training had a significant role in facilitating couplet care.

## Implementation practices in Sweden and Finland

In 1998, the level II neonatal unit of Västerås was the first unit in Sweden to adapt their facilities for couplet care. In 2003, the first unit providing couplet care for all admissions opened at Danderyd Hospital, Karolinska University Hospital in Stockholm [17]. Subsequently, couplet care has been increasingly implemented in both Sweden and Finland, especially over the last 10 years. Below we report on the current practices according to a survey on couplet care and single-family room design, carried out in May 2023.

### Sweden

**Fig. 4:**
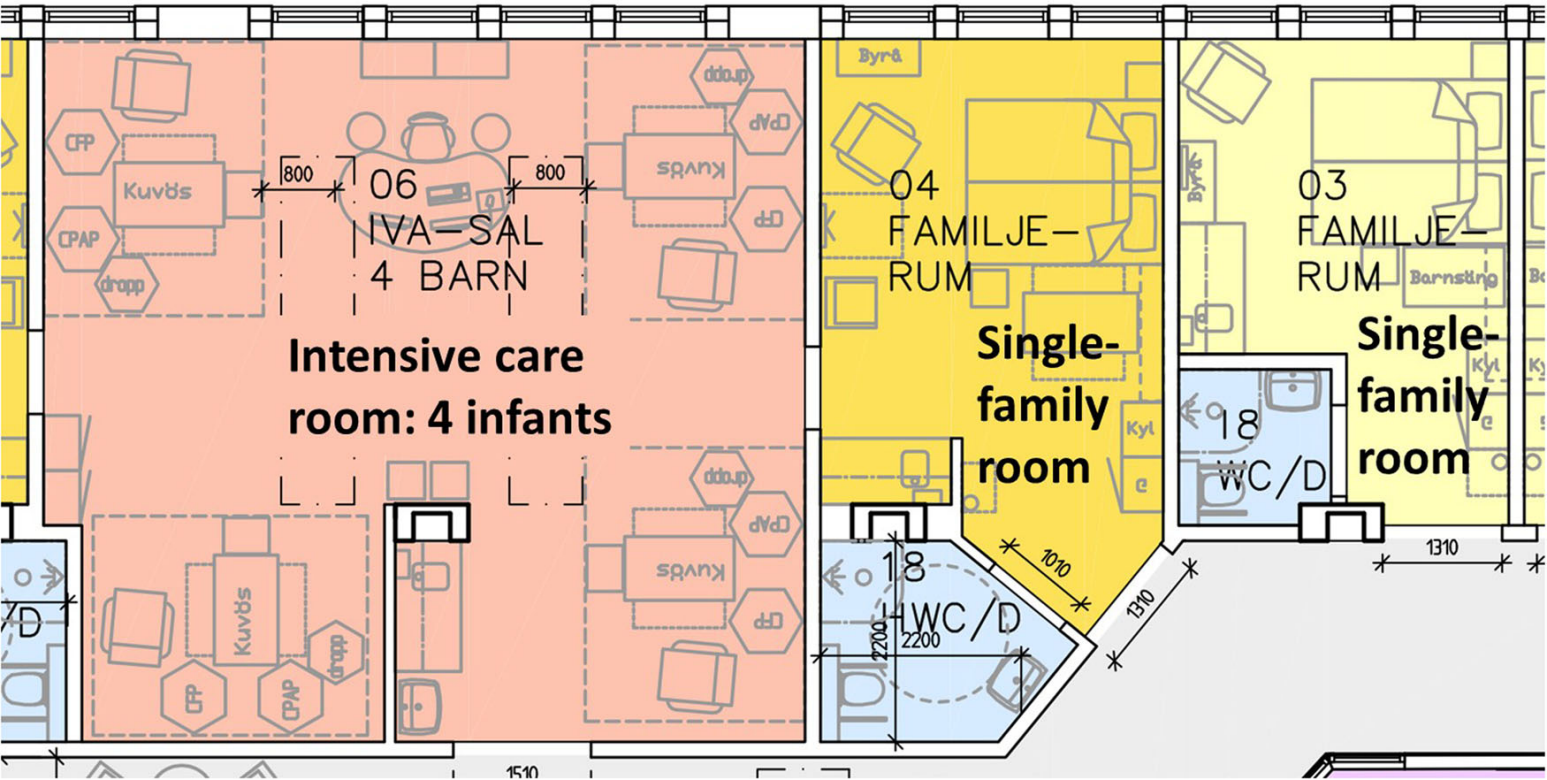
A room layout example from the neonatal intensive care unit at Danderyd, Karolinska University Hospital, Sweden. An open bay area for the intensive care of up to 4 infants, with space for a parent bed or reclining chairs by the infant ’s care space. Adjacent single-family rooms for the parents and infant once the infant ’s condition improves, or directly after birth for moderately sick or preterm infants and their parents. Some of the single-family rooms have larger shower bathrooms for mothers that need assistance. Architect: Snidare Arkitekter AB.

In Sweden, the development of couplet care has been founded on changing care culture through the systematic training of staff [54, 55]. Although a few hospitals implemented couplet care some 20 years ago, there has been a clear shift during the last few years towards non-separation, early skin-to-skin contact and providing couplet care also for sick and preterm infants. Sixteen of the 38 Swedish units report providing couplet care in the neonatal unit. Six of these units reported limiting couplet care to low-risk mothers and one reported to provide couplet care only for more stable infants and their low-risk mothers. Several hospitals have ongoing implementation processes and clear plans to start or expand couplet care and non-separation practices in the very near future, as well as to rebuild their neonatal units for improved facilities and an increased number of single-family rooms for intensive care. The design of most neonatal units in Sweden consists of a combination of single-family rooms and small open bay areas for two to four infants in need of intensive care. The open bay rooms have adjacent single-family rooms for the parents whose infants are in the intensive care room and for the parents and newborns in need of less intensive care (Fig. 4). All units reported providing non-separation through increasingly specialized care for term, or close to term, moderately ill newborn infants in the maternity unit, thus reducing the need for transfer to the neonatal ward. Supported by national governmental institutions, the Swedish Association of Local Authorities and Regions has established a working group to develop national guidelines for mother-newborn couplet care. The guidelines will include recommendations for hospital managements and a plan for follow-up of their compliance.

### Finland

**Fig. 5:**
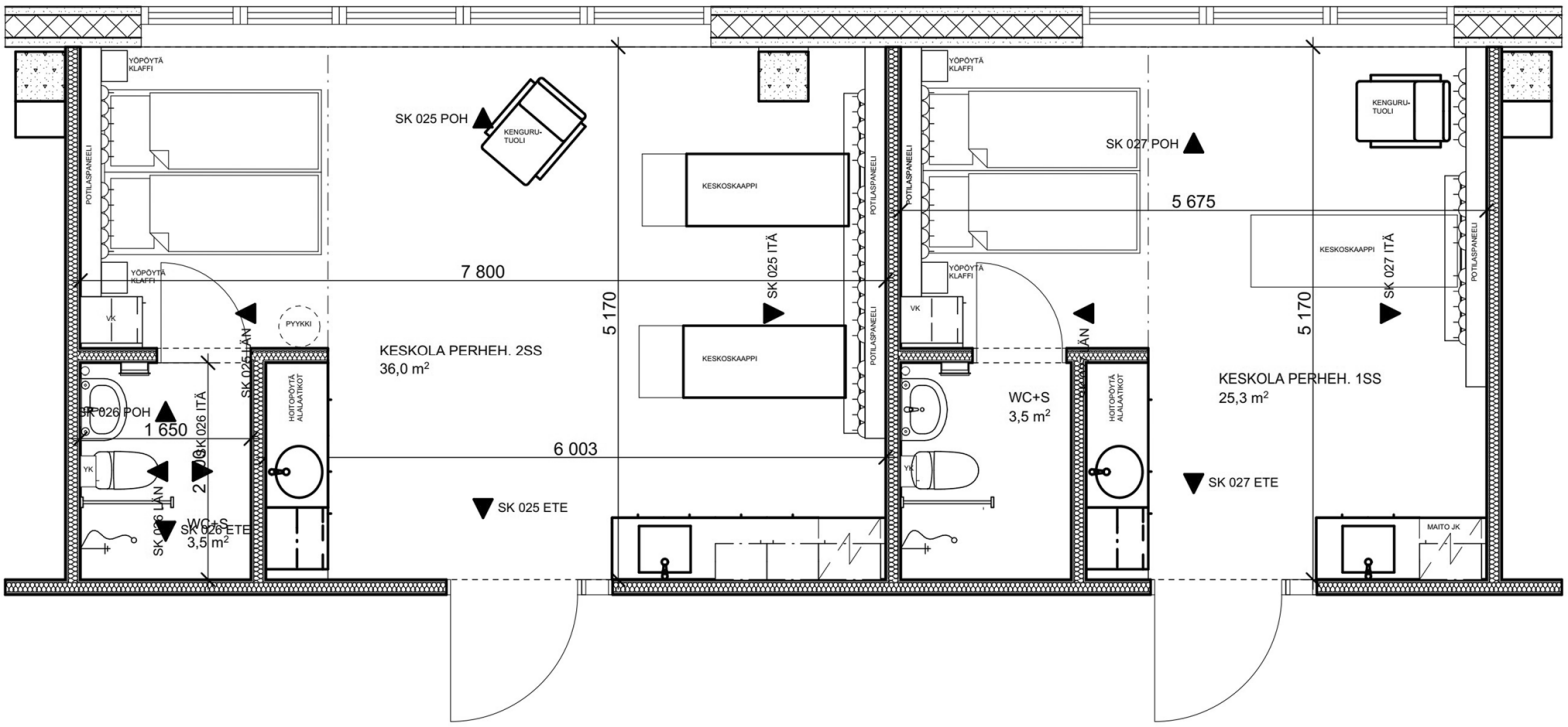
A room layout example from the neonatal intensive care unit at Turku University Hospital, Finland, with single-family room design for Mother-Newborn Couplet Care. On the left side, a room for twins which is used also for patients potentially needing onsite surgeries. On the right side, a room for a singleton without surgical needs. Architect group Reino Koivula.

Finland has 23 maternity hospitals. Eighteen of them reported that they provide couplet care in the neonatal intensive care unit. Seven of them provide couplet care for all mothers, eleven provide couplet care with limitations and five hospitals do not provide couplet care. Seven hospitals had limitations regarding the mother (e.g., couplet care only after vaginal birth), five hospitals had limitations regarding the infant (high-intensity care of the infant), and three hospitals had limited bed spaces for the mothers. Some hospitals report ongoing construction processes including clear plans to start or expand couplet care. Changes in both the care culture and interior architecture have been needed to implement couplet care in neonatal intensive care units in Finland. Care culture in the neonatal units has been changed through the systematic and structured training of the staff in Close Collaboration with Parents [52, 53]. At the same time, many hospitals have renovated their architecture. Old units have been renovated to include family rooms. Nine hospitals have rebuilt the whole unit using a single-family room design to accommodate mothers in the neonatal intensive care unit (Fig. 5). Five of them have only single-family rooms, and four of them have a separate procedure/intensive care room for occasional use. In our survey, only two hospitals in Finland did not have any single-family rooms. The change in architecture has had a major role in implementing couplet care as a routine practice in many hospitals in Finland.

## Vision and future direction

The vision is to keep and care for mothers and infants together from birth, irrespective of their clinical conditions, thus humanizing healthcare. Parents become change agents for their infant’s care and play a central role in the care of their infants. However, they need enabling facilities, a welcoming environment, supportive staff attitudes and interactions, information and support in practicing breastfeeding and skin-to-skin contact and practical and emotional support in caring for their infant. The meticulous implementation of couplet care for infants and their mothers facilitates safe care with improved quality and experience of care as well as improved short- and long-term outcomes for both. However, to support and scale-up the implementation of the concept of care that keeps mothers and newborns together more research is warranted. In summary, along with other essential evidence-based interventions, mother-newborn couplet care should be provided for every mother-newborn dyad with sick or preterm infants, to enhance survival, growth, health and development [38].
